# Laccase production and metabolic diversity among *Flammulina velutipes* strains

**DOI:** 10.1007/s11274-014-1769-y

**Published:** 2014-11-07

**Authors:** Grzegorz Janusz, Aleksandra Czuryło, Magdalena Frąc, Beata Rola, Justyna Sulej, Anna Pawlik, Marek Siwulski, Jerzy Rogalski

**Affiliations:** 1 Department of Biochemistry, Maria Curie-Skłodowska University, Akademicka 19 St., 20-033 Lublin, Poland; 2 Institute of Agrophysics, Polish Academy of Sciences, Doświadczalna 4 St., 20-290 Lublin, Poland; 3 Department of Vegetable Crops, Poznań University of Life Sciences, Dąbrowskiego 159 St., 60-594 Poznan, Poland

**Keywords:** Laccase, *Flammulina velutipes*, Metabolic diversity

## Abstract

Twelve *Flammulina velutipes* strains originating from Poland were identified using internal transcribed spacer (ITS) region sequencing. Based on the sequences obtained, the genomic relationship of the analyzed strains was determined. All *F. velutipes* strains were also characterized using Biolog FF MicroPlates to obtain data on C-substrate utilization and mitochondrial activity. The ability to decompose various substrates differed among the *F. velutipes* strains up to five times. The highest catabolic activities were characteristic for only two strains with capabilities to decompose up to 22 carbon sources. The correlation between carbon repression and laccase production by *F. velutipes* was analyzed based on glucose assimilation by these strains. Moreover, the influence of metal ions (Cu^2+^, Cd^2+^), veratric and ferulic acids, and temperature on laccase activities in the analyzed strains was determined. The results obtained proved that all the inducers influenced laccase expression in almost all the analyzed strains. However, the degree of induction depended not only on the strain used but also on the day of the induction.

## Introduction

Laccase belongs to a large and diverse family of blue multi-copper enzymes, which catalyze oxidation of a wide range of aromatic compounds using molecular oxygen as a terminal electron acceptor. It is one of the oldest known enzymes that was first described by Yoshida in 1883 and are widely distributed in nature (Giardina et al. 2010; Mayer and Staples 2002; Pardo et al. 2012). The enzyme has been detected in a variety of organisms such as fungi, plants, bacteria, and some insects. However, most of the laccases studied so far are of fungal origin. Laccase activity has been demonstrated in *Basidiomycetes*, *Ascomycetes*, and *Deuteromycetes* (Bourbonnais et al. 1995; Gedikli et al. 2010). Among them, white rot fungi belonging to *Basidiomycetes* are the best known laccase producers (Sadhasivam et al. 2008). Fungal laccases carry out a variety of roles including lignin degradation, pigment biosynthesis, fruiting body formation, detoxification, morphogenesis, as well as pathogenesis. Interestingly, due to its ability to oxidize substrates under alkaline conditions, the novel laccase isolated from the culture fluid of *Flammulina velutipes* could be used in hair coloring systems. Moreover, as an oxidase, laccase is used in many other agricultural, industrial, and medicinal applications e.g. as a catalyst for the industry of anti-cancer drugs (Couto and Herrera 2006; Lundell et al. 2010; Mayer and Staples 2002; Saito et al. 2012).

Due to its environmental, industrial, and potential biotechnological applications, laccase has been intensively studied since the 19th century. Studies of the structure of laccase-encoding genes and regulation of this extracellular lignin-modifying enzyme gene expression are very helpful for intensifying the productivity of native laccases in fungi and for a better insight into the physiological role in which different laccase isoforms are involved (Unyayar et al. 2006). Regulation of laccase-encoding gene expression is a complicated process influenced by a number of physiological factors. It is known that the expression of lignin-modifying laccases is affected by carbon and/or nitrogen sources as well as their concentration and ratio (Leatham and Kent Kirk 1983; Sulej et al. 2013). Many reports are focused on regulation of laccase expression by metals. Among the various metals (Cd^2+,^ Ag^2+^, Hg^2+^, Mn^2+^), copper is one of the well-known inducers of laccase activity (Baldrian and Gabriel 2002; Galhaup et al. 2002; Karahanian et al. 1998; Soden and Dobson 2003). The effect of Cu^2+^ on laccase synthesis can be explained at both the protein and transcriptional level. The active site of laccase contains four copper atoms and this can explain the action of copper on laccase activity. Cupric ions can be incorporated into the apoprotein, giving rise to the active enzyme (Larrondo et al. 2003; Makela et al. 2013). Lorenzo et al. (2006) observed that laccase activity measured in cultures grown in the presence of 3.5 mM Cu^2+^ was approximately 11-fold greater than that in cultures containing no copper. Transcriptional induction of laccase by copper has been shown in a number of white-rot fungi such as *Trametes versicolor*, *Ceriporiopsis subvermispora*, *Pleurotus ostreatus*, *P. sajor*-*caju*, *Coriolopsis rigida*, and *Trametes pubescens* (Piscitelli et al. 2011). Piscitelli et al. (2011) reported that laccase induction in various fungal species had been demonstrated in the presence of many different phenolic compounds, especially 2,5-xylidyne, veratryl alcohol, and ferulic acid. It is known that aromatic compounds tend to regulate ligninolytic enzyme synthesis although their effect is very specific depending on the physiological peculiarities of fungi (Elisashvili and Kachlishvili 2009). Since laccase synthesis is part of fungal metabolism and white rot fungi are often able to decompose several tree species, the diversity in laccase expression and metabolism among strains of the same species should be analyzed. One of the white rot fungi capable to degrade several tree species is *Flammulina velutipes* also known as the winter mushroom. It is an important edible fungus cultivated in many Asian countries. Thanks to presence of various bioactive compounds (polysaccharides, protein-glucan complexes, sterols, lectins, peroxidases, laccases, cellulases, and proteases), *F. velutipes* can be used in many medical, pharmaceutical, and industrial applications (Hassan et al. 2012). Moreover, a previous study has reported that *F. velutipes* belongs to the phylum *Basidiomycota* and is one of the white rot fungus capable of production of extracellular lignin-modifying laccase (Kim et al. 2013; Lee and Suh 1985; Otsuka Saito et al. 2013; Zhang et al. 2004). The aim of this paper was to demonstrate that closely related strains of *F. velutipes* might differ in laccase production as a response to commonly used inducers. Moreover, we attempted to show that the differences mentioned above might be related to the induction time. Strains diversity in laccase production was compared to the ability of *F. velutipes* to decompose 95 carbon sources. Finally, the paper proves that each strain requires a separate approach to optimization of culture conditions in order to boost up enzyme activities.

## Materials and methods

### Chemicals

Syringaldazine (4-hydroxy-3,5-dimethoxybenzaldehyde azine), ferulic acid (4-hydroxy-3-methoxycinnamic acid), and veratric acid (3,4-dimethoxybenzoic acid) were supplied by Sigma-Aldrich (St. Louis, MO, USA), while l-asparagine was purchased from Merck (Darmstadt, Germany). All other products used were of reagent or analytical grade and purchased locally.

### Strains and culture conditions

*Flammulina velutipes* strains FV1-FV12 were obtained from the culture collection of the Department of Vegetable Crops, Poznań University of Life Sciences. The fungi were maintained on 2 % (w/v) malt agar slants.

To obtain the inocula, pieces of agar were grown in the Lindenberg and Holm medium (Lindeberg and Holm 1952) in non-agitated conical flasks for 7 days at 28 °C. Seven-day-old mycelia were homogenized in a disperser homogenizer T18 basic ULTRA-TURRAX (IKA, Staufen, Germany). The fragmented mycelial culture (10 % v/v) was used as a standard inoculum for further studies. The shaken cultures were run up to 8 days at 28 °C in 24-well microplates (each well with 2 mL of the Lindeberg–Holm medium) placed in an orbital rotary shaker at 300 rpm. Each strain was run in four replications. The samples (140 μL) were collected every 24-h.

Putative laccase inducers were dissolved in water (Cu^2+^ and Cd^2+^) or in ethanol (veratric and ferulic acids) as stock solutions and sterilized by filtration through a Sterivex-GS filter unit (pore size, 0.22 μm; Millipore Corp.). These were added to the fungal cultures on the fourth or sixth day of incubation and their final concentrations in the optimized Lindeberg-Holm medium were 1.0 mM (veratric and ferulic acids), 10 μM (Cu^2+^), and 25 μM (Cd^2+^). The final concentration of ethanol in the growth medium was always less than 0.5 % and an equivalent amount of ethanol was added to control flasks without the aromatic inducer. The influence of low and high temperature was studied at 4 and 40 °C, respectively. The temperature induction was performed on day four or six by incubating the microplates for 2 h (Jarosz-Wilkolazka et al. 1998).

### Assays

Laccase activity in the culture fluid was measured spectrophotometrically at 525 nm in a Shimadzu UV–Vis 160A spectrophotometer (Tokyo, Japan) using syringaldazine as a substrate (Leonowicz and Grzywnowicz 1981). Enzyme and substrate blanks were included. One nano katal (nkat) of laccase activity was defined as the amount of enzyme catalyzing the production of one nano mol of the colored product (quinone, ε^M^ = 65,000 M^−1^ cm^−1^) per second at 25 °C and pH 5.5 and expressed as nano katals per litre of culture (nkat/L). The glucose concentration was determined by the Lloyd and Whelan method (Lloyd and Whelan 1969).

### Genomic DNA isolation and PCR amplification of the ITS region

The culture of *F. velutipes* was grown stationarily in the Lindeberg and Holm medium (Lindeberg and Holm 1952) at room temperature (25 °C) for 7 days. Mycelia were harvested through Miracloth (Merck, Whitehouse Station, NJ, USA), washed twice with TE buffer, and frozen in liquid nitrogen. DNA was isolated according to Borges et al. (1990). The purity and quantity of the DNA samples were evaluated using an ND-1000 spectrophotometer (Thermo Scientific, West Palm Beach, FL, USA). PCRs were performed using Sigma RedTaq in a T-personal thermal cycler (Biometra, Goettingen, Germany). To confirm the fungus identity, the ITS region in the nuclear ribosomal repeat unit was determined by direct sequencing of the PCR products amplified with ITS1-ITS4 primers as described previously (White et al. 1990) (Table 1). Automatic Sequencing was performed using a BigDye™ Terminator Cycle Sequencing Kit and an ABI PRISM 310 or ABI PRISM 3730 XL sequencer (Applied Biosystems, Carlsband, CA, USA).

**Table 1:** Gene-specific primer sequences and their annealing temperatures

Primer	Sequence 5′–3′	Tm (°C)
ITS1	TCCGTAGGTGAACCTGCGG	55.4
ITS4	TCCTCCGCTTATTGATATGC	49.7

### Bioinformatic tools

Sequencing data were analyzed with Lasergene v.8.0 software (DNASTAR, Inc). Database searches were performed with the BLAST and FASTA programs at the National Centre for Biotechnology Information (Bethesda, MD, USA) and the European Bioinformatic Institute (Hinxton, UK).

### Biolog microplate analysis

Carbon utilization and mitochondrial activity were investigated using Biolog FF MicroPlates (Biolog, Inc. USA). The FF MicroPlate test panel comprises 96 wells with different carbon-containing compounds and a control well. Nutrients and test reagents were pre-filled and dried into the 96 wells of the microplate. Tetrazolium violet (TV) was used as a redox dye to measure colorimetrically the mitochondrial activity resulting from oxidation of metabolizable carbon sources. All wells were colorless when first inoculated. In the current study, absorbance readings were monitored at 750 nm. The 750-nm reading measures turbidity reflecting mycelial growth and utilization of the test substrate. The substrate richness index was calculated to evaluate the number of substrates utilized by each strain during incubation time. Optical density higher than 0.25 for each substrate was considered as a positive response of the particular strain.

*Flammulina velutipes* strains were grown on 2 % (w/v) potato dextrose agar (PDA) under ambient laboratory conditions of diffuse daylight and temperature (25 °C). The inoculum was extracted after conidial maturation (7–14 days) with a sterile, wetted cotton swab over mycelium areas. The mycelium was suspended in 16 ml of a sterile phytagel solution (0.25 % phytagel, 0.03 % Tween 40) in disposable borosilicate test tubes (20 × 50 mm). The suspension was agitated in a vortex mixer for about 5 s, and additional inoculum was added as required to adjust the density of the suspension to 75(±2) % of transmittance using a turbidimeter (Biolog, USA). For preparation of each inoculum, the turbidimeter was blanked with an uninoculated inoculating fluid tube by adjusting the 100 % transmittance. 100 μL of the mycelial suspension at the desired turbidity were dispensed into each well of the Biolog FF MicroPlate (Biolog, Hayward, CA, USA). The inoculated microplates were incubated in the dark at 26 °C and the absorbance was determined after 24, 48, 72, 96, 120, 144, 168, 192, 216, and 336 h at 750 nm using a microplate reader. The most consistent readings came from the 336-hour-old Biolog plates and these were used in the analyses.

### Statistical analysis

The data obtained were joined in a single matrix and analyzed using STATISTICA 10.0 (StatSoft, Inc.) software package according to Druzhinina et al. (2006). Subsequently, all data were carefully examined towards mean, minimum, maximum, and standard deviation values and finally for outliers. To determine groups in the data set, cluster analysis (Hartigan 1975; Tryon 1939) was applied. By using this method, utilization of carbon substrate by the particular strain was determined. This analysis was helpful in identification of strains with similar carbon utilization profiles and simultaneous grouping both carbon sources and strains in a two-way joining analysis. In most of the results obtained, Euclidian distance allowed the cluster-joining analysis, whereas the greatest distance between any two objects in the different clusters was used to analyze complete linkage as distances between clusters. Substrate richness was analyzed with ANOVA, which as a result allowed concluding about strain growth on individual carbon sources.

## Results

### PCR amplification of the ITS region

All the analyzed strains were isolated in Poland (Table 2). These strains were identified at the species level by analysis of their ITS region. For each strain, one product ranging from 785 (FV1) to 807 bp (FV6) in length was obtained from PCR with ITS1-ITS4 primers and followed by direct sequencing. The complete sequences of these products indicated from 95 to 99 % identity to the *F. velutipes* ITS sequences. GenBank accession numbers assigned to the nucleotide sequences determined in this study are presented in Table 2.

**Table 2:** GenBank accession numbers of ITS sequences of *F. velutipes* strains determined in this study

Strain source/other collection^ab^	Host organism	Geographical origin	GenBank accession
FV1	*Fraxinus excelsior*	Wojnowice (Poland)	JX294499
FV2	*Acer negundo*	Poznań (Poland)	JX294500
FV3	*Acer negundo*	Poznań (Poland)	JX294501
FV4	*Populus alba*	Kiekrz (Poland)	JX294502
FV5	*Alnus glutinosa*	Rakoniewice (Poland)	JX294503
FV6	*Ulmus minor*	Jarocin (Poland)	JX294504
FV7	*Populus nigra*	Jarocin (Poland)	JX294505
FV8	*Salix caprea*	Zielona Góra (Poland)	JX294506
FV9	*Alnus incana*	Wojnowo (Poland)	JX294507
FV10	*Acer platanoides*	Tychy (Poland)	JX294508
FV11	*Fagus sylvatica*	Poznań (Poland)	JX294509
FV12	*Populus nigra*	Rakoniewice (Poland)	JX294510

^a^FCL, Fungal Collection of Lublin, Department of Biochemistry, Maria Curie-Sklodowska University, Lublin, Poland

^b^ULSP, Department of Vegetable Crops, University of Life Sciences, Poznan, Poland

**Fig. 1:**
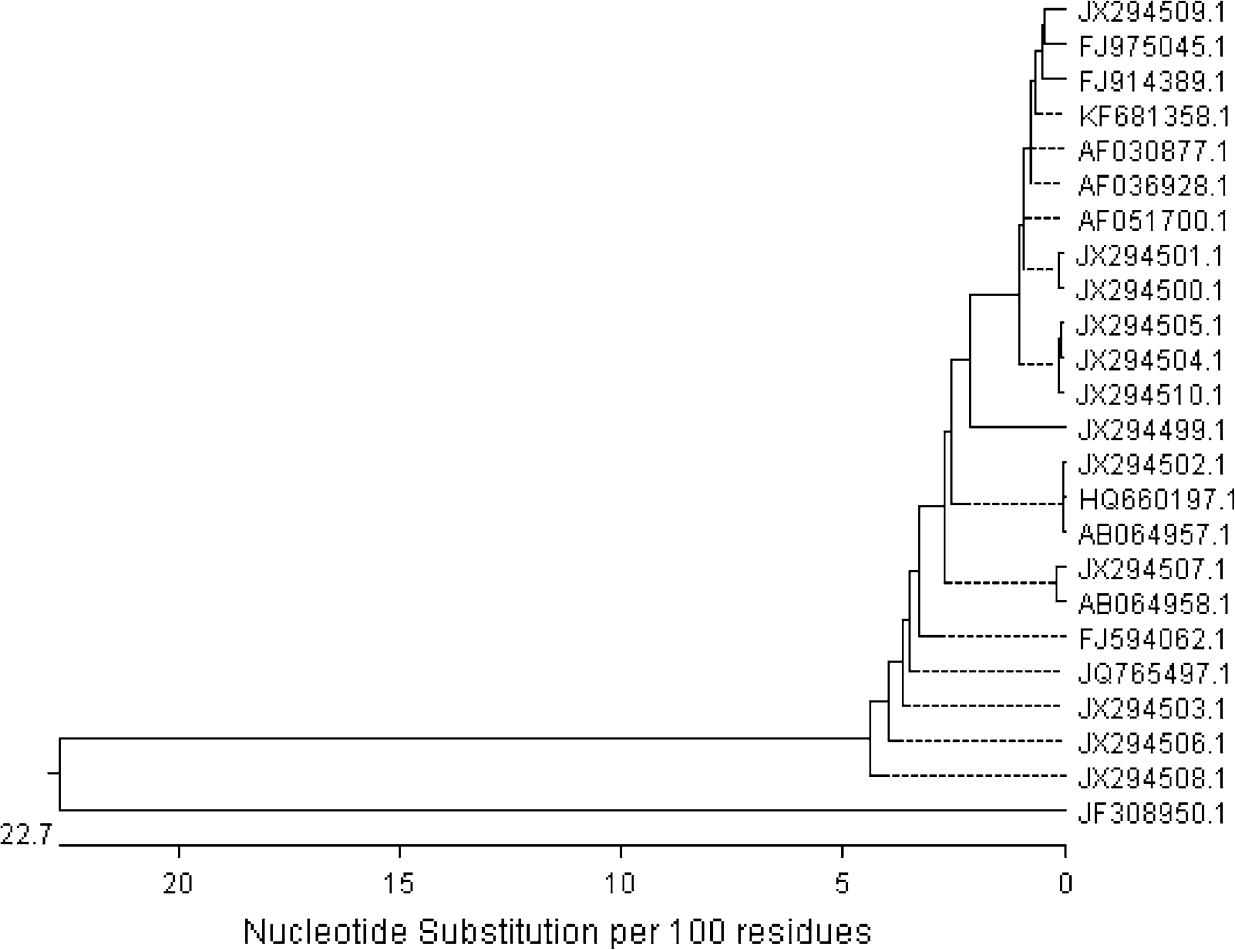
Cluster analysis-based dendrograms constructed with: 22 ITS sequences of *F. velutipes* generated by neighbor-joined method using the MegAlign, Lasergene (DNASTAR) software. *F. velutipes*: JX294509, FV11; FJ975045, SLO F-1594; FJ914389, SLO F-1593; KF681358, F_v18S; AF030877, 7200; AF036928; CBS771.81; AF051700, Olexia; JX294501, FV3; JX294500, FV2; JX294505, FV7; JX294504, FV6; JX294510, FV12; JX294499, FV1; JX294502, FV4; HQ660197, no strain number; AB064957, MH09210; JX294507, FV9; AB064958, MH09236; FJ594062, FCL251; JQ765497, Jin Zhen; JX294503, FV5; JX294506, FV8; JX294508, FV10. *T. versicolor* ITS sequence (JF308950) was used as out group

The genomic relationship between the studied *Flammulina* strains and ten most similar strains according to BLAST analysis are presented on the dendrogram constructed with an unweighted pair group method with arithmetic means (UPGMA) cluster analysis (Fig. 1). Based on the data from the ITS sequences, all the 12 *Flammulina* strains did not form a monophyletic group and were classified into several clusters with other *F. velutipes.* The analyzed strains shared profile similarity of 97.2 % with exception of FV1, which displayed a lower identity level −95.4 %. However, this strain seems to be more similar to other *F. velutipes* strains with profile similarity from 96.5 %.

### Biolog microplate analysis

**Fig. 2:**
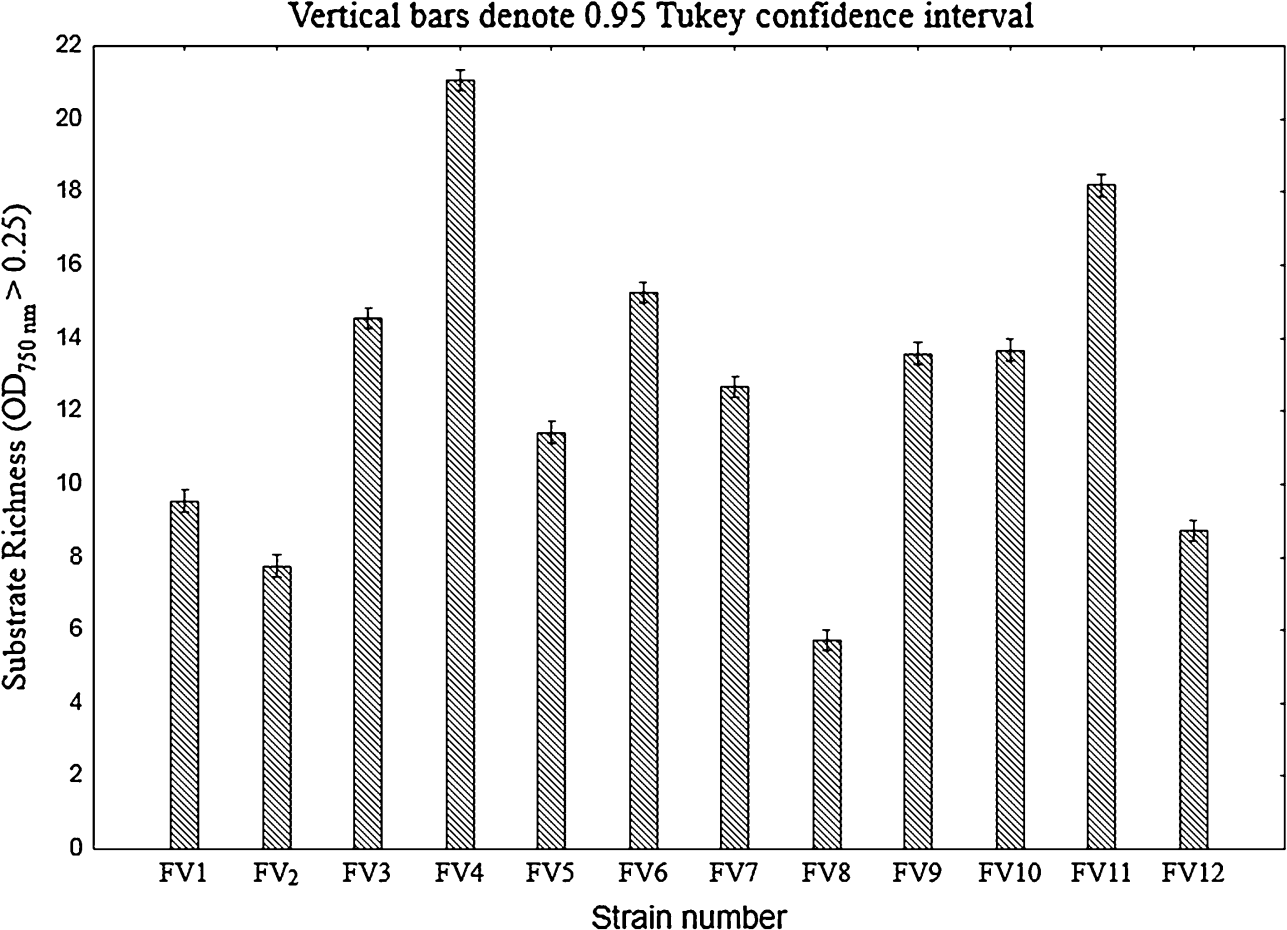
Substrate richness utilized by *F. velutipes* strains

**Fig. 3:**
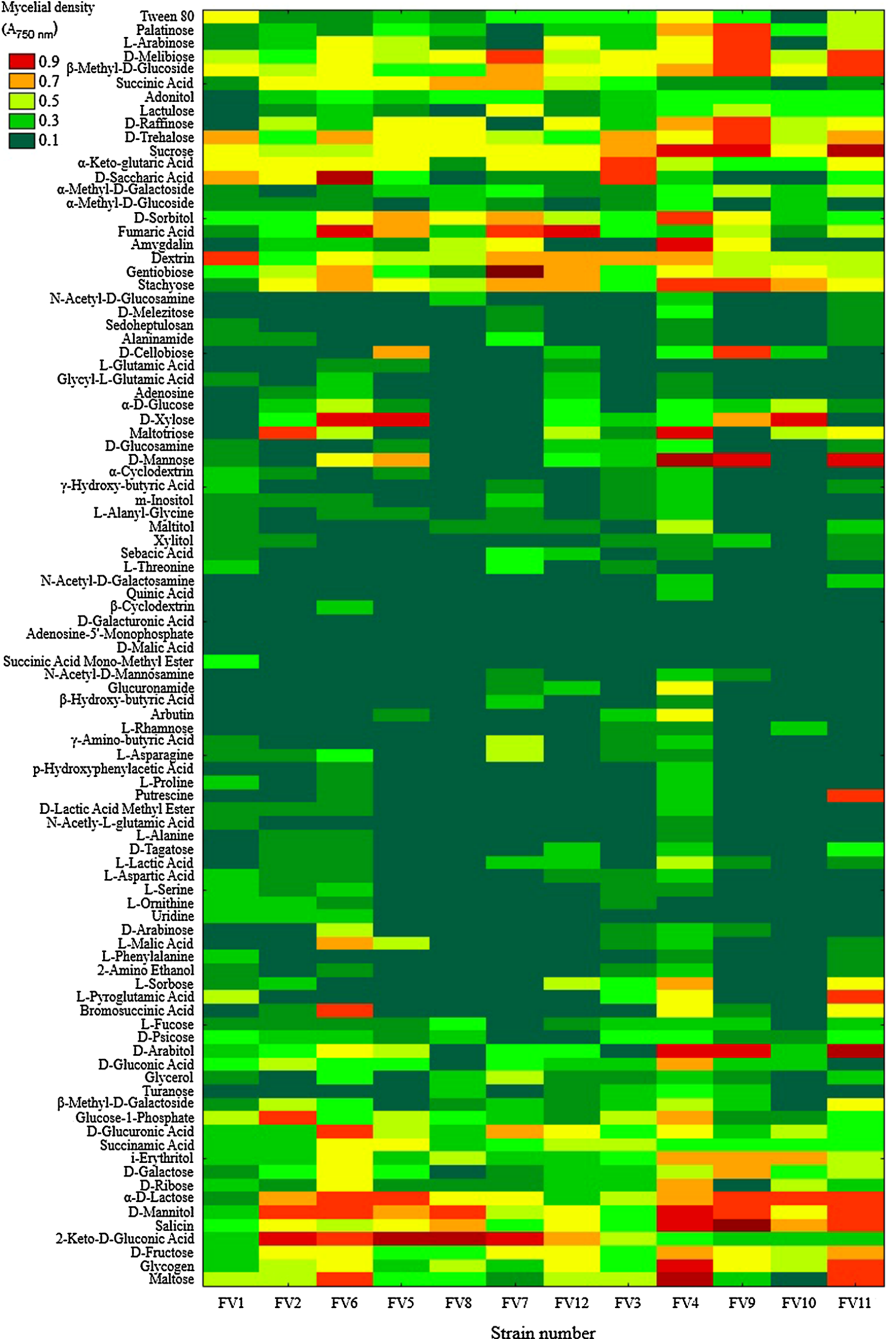
The heat map of carbon utilization patterns of *F. velutipes* strains

**Fig. 4:**
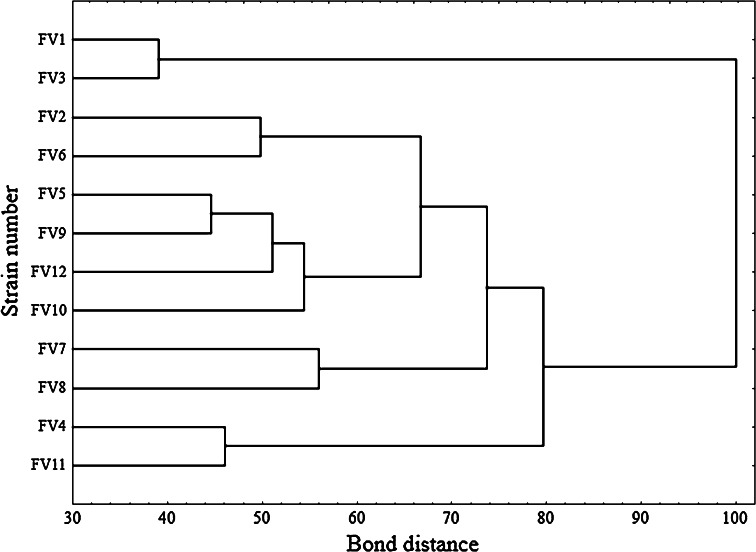
Cluster analysis-based dendrograms showing diversity of selected *F. velutipes* strains with respect to utilization of carbon sources from a FF-MicroPlate (Biolog Inc.)

The 12 *F. velutipes* strains were also characterized using Biolog FF MicroPlates to obtain data on C-substrate utilization and mitochondrial activity. The metabolic activity of the analyzed strains (AWCD—average well color development) was calculated based on absorbance at 750 nm (Fig. 2). Clearly, this value differed among the *F. velutipes* strains up to five times. The highest catabolic activities were exhibited by strains FV4 and FV11 with capabilities to decompose 22 and 12 carbon sources, respectively. In turn, FV8 was able to assimilate only 5 and FV2 up to 9 C-sources. It may be noticed in Fig. 3 that the carbohydrates constitute a group of the most easily metabolized carbon sources by all the analyzed *F. velutipes* strains. In contrast, amino acids are hardly decomposed by these fungi and only FV1 and FV3 may be distinguished as capable of the decomposition. Surprisingly, l-pyroglutamic acid is the most easily decomposable source but only by four strains, i.e. FV1, FV3, FV4, and FV11. The dendrogram analysis (Fig. 4) resulted in formation of two clusters. The first one comprises most of the strains (FV2 and FV4-FV12), which were able to decompose carbon sources from all the groups. The second cluster consists of FV1 and FV3, which were able to decompose mainly amino acids.

### Laccase production

**Fig. 5:**
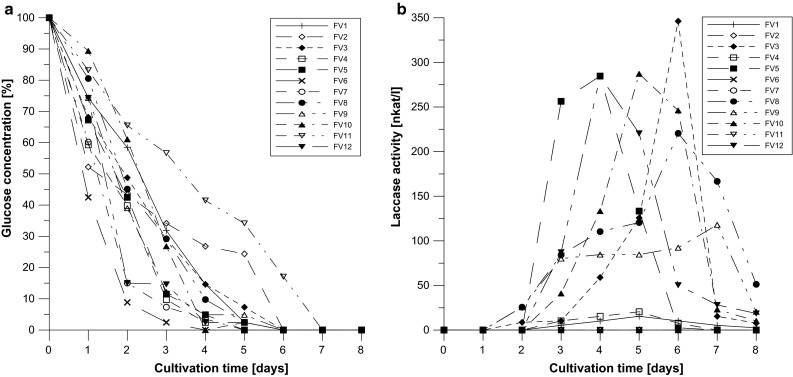
Glucose assimilation (**a**) and laccase production (**b**) by *F. velutipes* strains

The correlation between carbon repression and laccase production by *F. velutipes* was analyzed based on glucose assimilation by these strains using the Lloyd–Whelan method (Fig. 5a) in comparison to laccase activities. In a majority of the analyzed strains, the glucose concentration decreased to 10–15 % on the fourth day. To analyze possible carbon repression as typically inducing laccase activity, day 4 and 6 were selected for the experiments concerning the influence of other factors. Not all of the analyzed strains were able to produce laccase without the inducers. In the control experiments, the highest activities were noted for FV10 (day 5), FV5 and FV12 (day 4), and FV3 and FV8 (day 6). The other strains barely produced laccase or the activities were not detected (Fig. 5b).

**Fig. 6:**
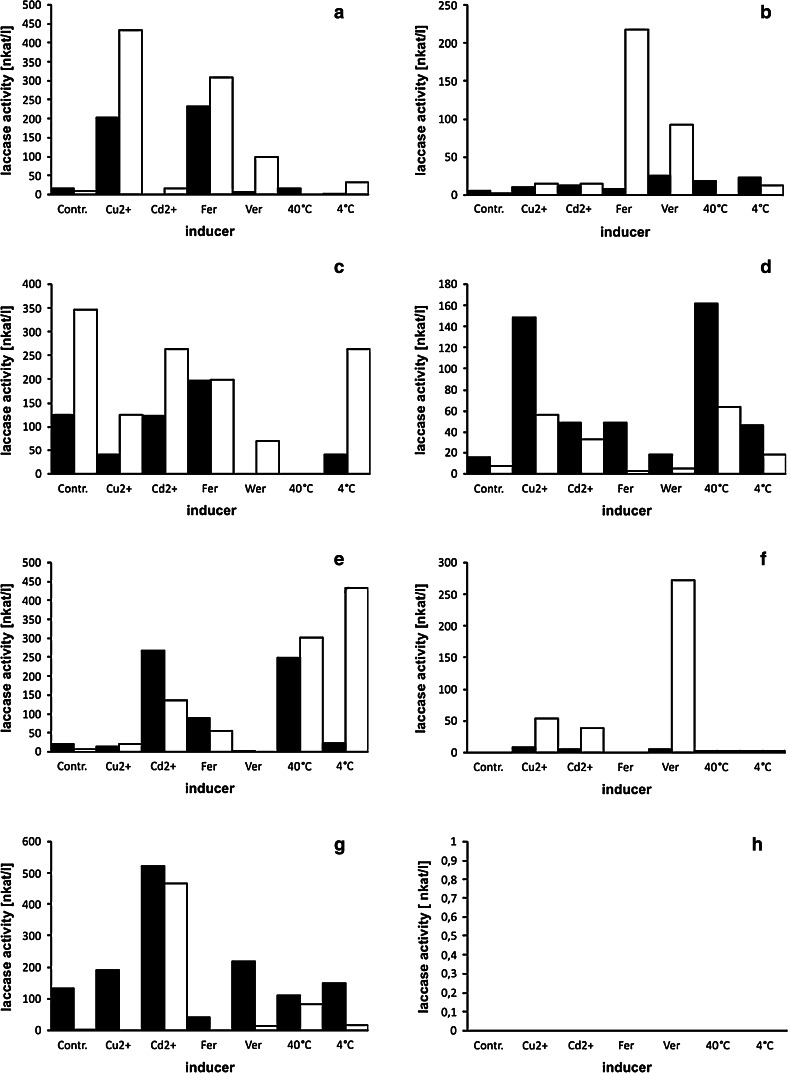
Influence of inducers on laccase activity in *F. velutipes* cultures (**a**, **b** strain FV1; **c**, **d** strain FV3; **e**, **f** strain FV4; **g**, **h** strain FV5)—induction on the day 4th (*left column*), on the day 6th (*right column*). Enzyme activities after 24 h are *black boxed*, activities after 48 h are *white boxes*. X-axis descriptions—inducers: Cu^2+^, copper ions; Cd^2+^, cadmium ions; Fer, ferulic acid; Ver, veratric acid; 40 °C, culture temperature elevated to 40 °C; 4 °C, culture temperature lowered to 4 °C

**Fig. 7:**
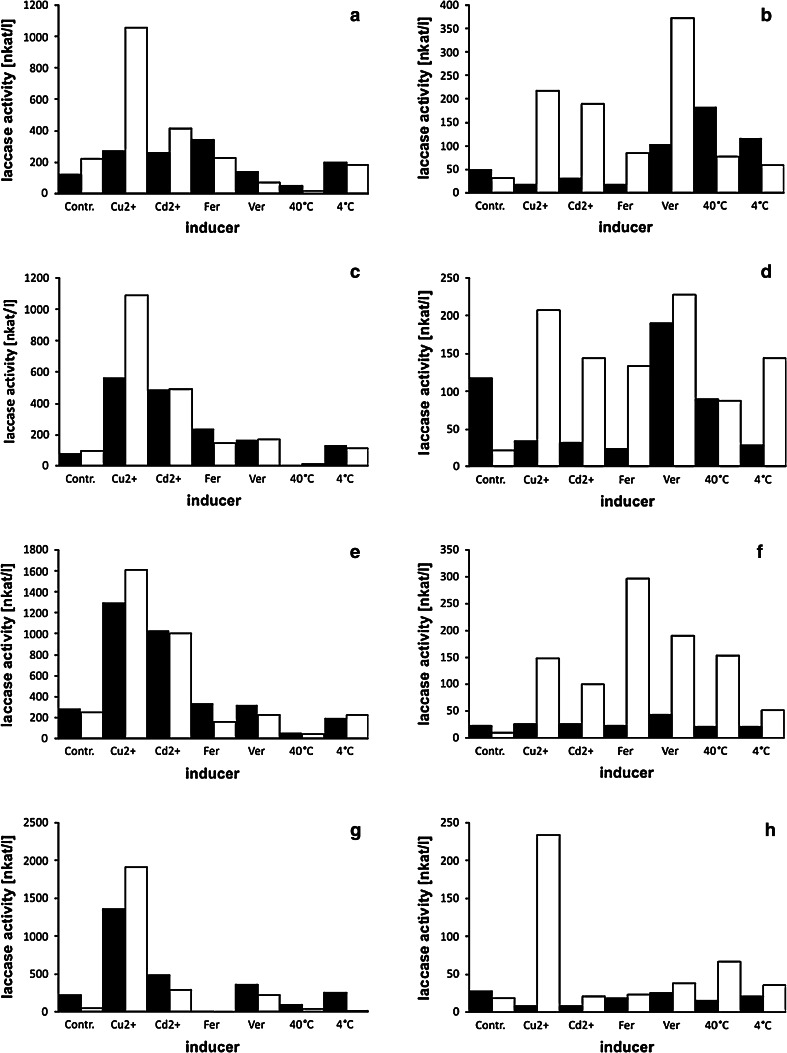
Influence of inducers on laccase activity in *F. velutipes* cultures (**a**, **b** strain FV8; **c**, **d** strain FV9; **e**, **f** strain FV10; **g**, **h** strain FV12)—induction on the day 4th (*left column*), on the day 6th (*right column*). Enzyme activities after 24 h are *black boxed*, activities after 48 h are *white boxes*. X-axis descriptions—inducers: Cu^2+^, copper ions; Cd^2+^, cadmium ions; Fer, ferulic acid; Ver, veratric acid; 40 °C, culture temperature elevated to 40 °C; 4 °C, culture temperature lowered to 4 °C

In order to analyze the influence of the inducers on laccase production by *F. velutipes* strains, the chosen factors were introduced to the fungal cultures on the 4th and 6th day (Figs. 6, 7). The results obtained proved that all the inducers influenced laccase expression in almost all the analyzed strains. However, the degree of induction was dependent not only on the strain used but also on the day of the induction. Copper ions appeared to induce laccase production in a number of *F. velutipes* strains (FV1, FV3, FV4, FV8, FV9, FV10, FV12). It should be noted that strain FV12 produced laccase only in cultures supplemented with Cu^2+^, which resulted in a 38-fold increase in enzyme activities after 48 h. Cadmium was proved to induce laccase production in the FV3, FV4, FV5, FV8, FV9, and FV10 strains; however, the highest laccase activities appeared in cultures of FV5 with a 182-fold increase in comparison to the control experiment. The most vulnerable strain to induction by ferulic and veratric acids was FV1, which secreted laccase with an over 85-fold (ferulic acid) and 36-fold (veratric acid) increase. It should be noted that this strain barely produced laccase in the control experiments. In turn, strain FV4 proved to be a laccase producer only after induction with veratric acid (a 270-fold increase in laccase activities). Bearing in mind that wild *F. velutipes* produces fruiting bodies from November till even March and that laccase is often engaged in this process, the influence of low temperature (4 °C) on laccase activities was tested. Surprisingly, only the FV4 and FV9 strains appeared to be induced by lowering the culture temperature; however, laccase activities increased up to 56-times. Higher culture temperature (40 °C) increased laccase activities in strains FV3, FV4, FV8, FV9, FV10, but the results obtained were not as spectacular as in the case of induction with other factors reaching maximally a 39-fold increase in comparison to the control experiments.

## Discussion

Almost all the fungi examined till now secrete more than one laccase isozyme and, moreover, their production is regulated differentially by a number of environmental factors (Piscitelli et al. 2011). Therefore, analysis of the influence of a single inducer on laccase isozyme synthesis is often shaped by medium composition or culture conditions. As this multicopper oxidase production is often triggered by depletion of carbon or nitrogen sources (Janusz et al. 2013), it is almost impossible to observe an exclusive influence of other factors such as metals or xenobiotics even in laboratory conditions. Bearing in mind that all enzyme isoforms are assessed simultaneously by spectrophotometric measurement of laccase activity, we have chosen *F. velutipes*, which was proved by electrophoresis to produce one form of laccase (data not shown). Moreover, all the twelve strains were isolated from the different tree hosts in western Poland near Poznań (possibly narrow area) to enable analysis of metabolic and laccase regulation diversity (Table 2). Taxonomical identity of the isolated strains was proved by ITS analysis and the genomic relationship was determined based on the sequences obtained. This fact leads to a conclusion that, despite close geographical origin, the isolates may be regarded as separate strains (Fig. 1). Badalyan et al. (2006) proved that in Europe (where original diversity has been extirpated), there may exist a subset of Armenian *F. velutipes*, which is more diverse than those in Eurasia. Nevertheless, the most diverse Fv1 strain was the oldest in the culture collection—isolated in 1989—10 years before the other ones. It was already proved that laboratory strains may differ genetically from “younger” isolates or completely wild ones (Pawlik et al. 2012; Urbanelli et al. 2007). In consequence, the difference between Fv1 and Fv6 (22 bp) may result not only from the gene diversity. Similar results were obtained by Lindner and Banik (2011), who proved that environmental fungal ITS sequences are grouped by 95–98 % similarity within the *Laetiporus* genus. The above results may be supported by the thesis proposed by Schoch et al. (2012), who claim that intragenomic variation in the fungal genome may result from multiple paralogous or nonorthologous copies within single fruiting bodies or within axenic cultures. Moreover, the Biolog FF MicroPlates analysis proved that all the strains might be considered as individuals due to metabolic differences (Fig. 4). The discrepancies observed between the trees in Figs. 1 and 4 result from different features analyzed. ITS analysis allows a conclusion about the diversity of a single gene, whereas phenotypic characteristics performed with the Biolog system allow comparison of a number of enzymes engaged in fungal ability to decompose 95 substrates.

Considering the fact that laccase production should be treated as an important part of fungal metabolism, Biolog FF MicroPlates analysis was performed to asses *F. velutipes* ability to decompose various substrates. Using this method, the metabolic diversity of *Trichoderma* sp. isolated in South-East Asia has already been proved (Kubicek et al. 2003); however, it should be mentioned that molds are believed to be able to adapt more easily to changing environmental factors than white rot fungi. To our knowledge, this is one of the first such complex approaches to differentiate the metabolic diversity of wood degrading fungi. It seems astonishing that wood decomposing fungi may face obstacles in polysaccharide metabolism and prefer amino acids than sugars (FV1, FV3). Our initial Biolog experiments proved that these strains were even unable to grow only on glucose, and these results are in contrast to the growth analysis (Fig. 5) on the Lindeberg and Holm (1952) medium containing glucose as a carbon source. It is possible that these strains require other medium components to assimilate glucose. Moreover, molds seem to be better adapted evolutionarily to grow on single substrates than rotting fungi decomposing plant material consisting of complex polymers. According to Le Crom et al. (2009), the ability of *Trichoderma reesei* to assimilate glucose, fructose, mannose, N-acetylglucosamine and trehalose, d-xylose, d-arabinitol, mannitol, and the ß-linked disaccharides gentiobiose and cellobiose may be linked with higher production of cellulases, whereas utilization of α-linked sugars as glycogen may evidence lower production of this group of enzymes. Revankar and Lele (2006) observed a threefold increase in laccase production in *T. versicolor* when glucose was used as a carbon source in place of fructose. Interestingly, the carbon catabolite repression phenotype may be associated with a mutation in the *creA*/*cre1* gene, which encodes a C_2_H_2_-zinc finger-containing transcriptional repressor also up-regulating laccase synthesis (Atanasova and Druzhinina 2010; Piscitelli et al. 2011). It ensures utilization of glucose and some other carbon sources in preference to a large variety of other carbon compounds, which are far more complicated to decompose and require extracellular proteins. It seems that the capability of glucose assimilation remains unchanged whereas carbon catabolite-derepressed mutants appear to catabolize other sources more efficiently (Atanasova and Druzhinina 2010). Among the different carbon sources that were tested for laccase production, glucose showed the highest potential for the production of this lignin-modifying enzyme in many fungal strains. Periasamy and Palvannan (2010) showed a similar effect of increasing laccase activity in *P. ostreatus* in the presence of glucose compared to other carbon sources used in culture medium. However, it should be noted that despite the ability to consume glucose by FV 6 and 7 in comparison to FV 2 and FV 11, all four strains failed to produce extracellular laccase. In conclusion, this enzyme production is triggered not only by carbon depletion, but also by nitrogen deficiency and/or influenced by the relative concentration of the carbon and nitrogen source. Moreover, in the regulatory sites in the laccase promoter region are often multiplicated and more than one (responsible for carbon or nitrogen regulation) was identified, for example creA and Mig (carbon) or NIT2 (nitrogen) (Janusz et al. 2013). Almost every laccase promoter region brings up to light new insight into the regulation of this enzyme. However, in the available *F. velutipes* laccase promoter regions, the regulatory sites mentioned above have not been localized yet (Kim et al. 2013). It should be mentioned that many fungi produce not only extracellular but also intracellular laccase (Nagai et al. 2003; Xu et al. 2012), which raises the possibility that those strains unable to secrete extracellular laccase may produce an intracellular one.

FV1 and FV3 may assimilate amino acids as a nitrogen source and, therefore, their laccase production is triggered by nitrogen depletion in the medium. Lenin et al. (2002) showed that *Phanerochaete chrysosporium* laccase activity was suppressed by a high nutrient nitrogen concentration in the medium. On the other hand, expression of the laccase gene in *Trametes trogii* and *T. versicolor* is stimulated by nitrogen sources (Revankar and Lele 2006; Soden and Dobson 2001). Interestingly, laccase genes in several fungal species such as *Pleurotus sajor*-*caju* are differentially regulated at the transcriptional level by nitrogen sources. Four laccase genes have been found in *Pleurotus sajor*-*caju* but expression of only two of them, *lac 2* and *lac 4*, is stimulated by nitrogen sources (Soden and Dobson 2001). Sometimes even if the composition of the medium (wheat bran with the sawdust) boosts up laccase activities, it does not allow concluding how the enzyme synthesis is regulated (Sharma et al. 2008). Moreover, the authors mentioned above proved that a medium reach in amino acids inhibited laccase production by *F. velutipes*.

Until recently, many papers have indicated that laccase genes differ among fungal species and strains (Castilho et al. 2009; Chen et al. 2011; Ito-Kuwa et al. 2008; Kellner et al. 2007; Lyons et al. 2003). It is clear that the same diversity may be observed in laccase promoter regions among species and strains. Moreover, it has already been proved that laccase production is regulated by a number of environmental factors such as carbon and nitrogen sources, metals, xenobiotics, and temperature, and differential regulation is possible in the case of a number of laccase genes (Sulej et al. 2013). Therefore, it is not surprising that there are differences among strains not only in the ability to produce laccase but also in the influence of the environmental factors on production thereof. Only a few papers have indicated that strains of the same species may differ in laccase production rates, depending on the carbon source (Elisashvili and Kachlishvili 2009). Given the metabolic diversity among the *F. velutipes* strains, the influence of several environmental factors on laccase production was analyzed. Despite the fact that only few strains were analyzed, the degree of variation in laccase expression among the *Flammulina* strains is remarkable. Among the analyzed strains, we found those unable to produce this multicopper oxidase in the conditions used and strains with promising oxidoreductases activities, which in future may even be sources of a purified enzyme. It has already been demonstrated that fungi growing in the same habitat (laboratory conditions) may undergo loss of genetic diversity, which in consequence implies losing some genes (Pawlik et al. 2012; Urbanelli et al. 2007). There is a chance that the same process may be observed in nature. The chosen induction times have supported the thesis that laccase production is regulated by a number of factors acting in a synergistic or antagonistic way (Baldrian and Gabriel 2002; Faraco et al. 2003; Manubens et al. 2007; Periasamy and Palvannan 2010; Piscitelli et al. 2011). The higher activities after induction on the 4th day in comparison to the 6th day may be a consequence of synergistic action of the inducer and carbon repression or the physiological state of the mycelium. The ability to produce laccase under the influence of environmental factors in most of the analyzed strains differs between the 4th and 6th day not only in the rate of enzyme activities but also in the inducer pattern. However, it should be mentioned that the induced laccase activities were observed mostly 48 h after induction, which was proved earlier in *P. ostreatus* (Galhaup and Haltrich 2001) and *Cerrena unicolor* (Janusz et al. 2007).

The results obtained support the hypothesis about the metabolic diversity (also lignin metabolism) among strains belonging to the same white rot fungal species, especially with the ability to grow on different trees. Our results show that every fungal strain should be analyzed separately as an enzyme producer and that metabolic differences among strains are greater than expected. Moreover, it seems that many analyses using different media and conditions are required to define species as an only laccase or peroxidase producer, which may be nowadays facilitated by NGS techniques (Vanden Wymelenberg et al. 2009; Wang et al. 2013), especially by the analysis of fungal growth in a natural habitat.
